# Health-related quality of life in mild-to-moderate COVID-19 in the UK: a cross-sectional study from pre- to post-infection

**DOI:** 10.1186/s12955-024-02230-5

**Published:** 2024-01-30

**Authors:** Ioana-Alexandra Soare, Wajeeha Ansari, Jennifer L. Nguyen, Diana Mendes, Waqas Ahmed, Joanna Atkinson, Amie Scott, Jessica E. Atwell, Louise Longworth, Frauke Becker

**Affiliations:** 1 PHMR Limited, Ashby Business Park, Nottingham Road, LE651NG Ashby-De-La-Zouch, UK; 2 Pfizer Inc, New York, USA; 3 Pfizer Ltd, Tadworth, UK

**Keywords:** COVID-19, Health-related quality of life (HRQoL), EQ-5D-5L

## Abstract

**Background:**

The aim of this study was to estimate the impact of mild-to-moderate COVID-19 on health-related quality of life (HRQoL) over time among individuals in the United Kingdom, adding to the evidence base that had focussed on severe COVID-19.

**Methods:**

A bespoke online survey was administered to individuals who self-reported a positive COVID-19 test. An amended version of a validated generic HRQoL instrument (EQ-5D-5L) was used to measure HRQoL retrospectively at different timepoints over the course of an infection: pre-COVID-19, acute COVID-19, and long COVID. In addition, HRQoL post-COVID-19 was captured by the original EQ-5D-5L questionnaire. A mixed-effects model was used to estimate changes in HRQoL over time, adjusted for a range of variables correlated with HRQoL.

**Results:**

The study recruited 406 participants: (i) 300 adults and 53 adolescents with mild-to-moderate COVID-19 who had not been hospitalised for COVID-19 during acute COVID-19, and (ii) 53 adults who had been hospitalised for COVID-19 in the acute phase and who had been recruited for validation purposes. Data were collected between January and April 2022. Among participants included in the base-case analysis, EQ-5D-5L utility scores were lower during both acute COVID-19 (β=-0.080, *p* = 0.001) and long COVID (β=-0.072, *p* < 0.001) compared to pre COVID-19. In addition, EQ-5D-5L utility scores post-COVID-19 were found to be similar to the EQ-5D-5L utility scores before COVID-19, including for patients who had been hospitalised for COVID-19 during the acute phase or for those who had experienced long COVID. Moreover, being hospitalised in the acute phase was associated with additional utility decrements during both acute COVID-19 (β=-0.147, *p* = 0.026) and long (β=-0.186, *p* < 0.001) COVID.

**Conclusion:**

Patients perceived their HRQoL to have varied significantly over the course of a mild-to-moderate COVID-19 infection. However, HRQoL was found to return to pre-COVID-19 levels, even for patients who had been hospitalised for COVID-19 during the acute phase or for those who had experienced long COVID.

**Supplementary Information:**

The online version contains supplementary material available at 10.1186/s12955-024-02230-5.

## Key points

Health-related quality of life was significantly lower during the acute and long COVID-19 phases compared to before COVID-19.

Results suggested that health-related quality of life returned to pre-COVID-19 levels, even for patients who had been hospitalized for COVID-19 during the acute phase or for those who had experienced long COVID.

Symptoms specific to the acute COVID-19 phase were found to be associated with additionally lower health-related quality of life.

**Supplementary Information:**

The online version contains supplementary material available at 10.1186/s12955-024-02230-5.

## Introduction

Coronavirus disease 2019 (COVID-19) is an infectious disease caused by SARS-CoV-2 [1, 2]. In the United Kingdom (UK), almost 23 million cases have been reported as of June 2022, with the total number of deaths estimated at 181,093 [3].

Signs and symptoms of COVID-19 vary, ranging from mild respiratory tract illness similar to flu-like infections, to severe progressive pneumonia, multiorgan failure, and death. Common symptoms include fever, persistent dry cough, fatigue, shortness of breath, body ache, headache, loss of smell and taste, sore throat, and chest pain [4]. Evidence suggests that among unvaccinated patients at the beginning of the pandemic, i.e., before even the Alpha variant was designated as a variant of concern in December 2020, around 80% of infections were mild or asymptomatic, 15% were severe (requiring oxygen support), and 5% were critical (requiring ventilation) [5].

As of 31st July 2022, an estimated 2 million UK patients are self-reporting to experience longer-term symptoms, known as long COVID-19 or post-acute COVID-19 syndrome, following either a mild acute COVID-19 disease or more severe forms [6–8]. In the UK, the most common symptoms reported as part of long COVID include fatigue (62%), shortness of breath (37%), difficulty concentrating (33%) and muscle ache (31%) [8].

However, evidence capturing patients’ health-related quality of life (HRQoL) during both acute and long COVID, respectively, is limited. Most available evidence focuses on the impact of COVID-19 on the HRQoL of patients who had been hospitalised due to COVID-19 [9, 10] whereas evidence of mild-to-moderate COVID-19 on HRQoL is currently lacking.

The aim of this study was to capture how HRQoL changes over time among individuals with mild-to-moderate COVID-19 in the UK.

## Methods

### Study sample

Adults (aged 16 years or older) and adolescents (aged 12 to 15 years) from the UK were recruited via online panels and advertising campaigns on social media, respectively, by a recruitment agency (Global Perspectives). Adults were contacted by email and were provided with the link to the online survey. Upon entering the survey, they were presented with an information sheet and had to give their consent to participate in the study before being able to continue. Adolescents received the link to the survey only after their parents/carers/legal guardians had given consent by phone and had confirmed the adolescent’s eligibility to participate in the study. Upon survey completion, all participants were compensated with GBP 30.

Inclusion criteria required all respondents to self-report a positive COVID-19 test result from between four weeks and up to 12 months prior to the completion of the online survey. In addition, to ensure respondents suffered from mild-to-moderateCOVID-19, and not severe COVID-19, they were required to have received treatment or monitoring for COVID-19 in an outpatient setting only during the first four weeks after a positive COVID-19 test. However, a subgroup of patients who had been hospitalised for COVID-19 during the first four weeks after a positive COVID-19 test were also recruited as a validation sample to assess the robustness of our results and allow comparison of our findings to the previously published evidence on the HRQoL of patients hospitalised due to severe COVID-19. Details on inclusion and exclusion criteria on different subgroups of respondents can be found in the supplementary material (Table S1).

Data were collected between January and April 2022 through an online platform (Qualtrics) and were stored on a secure server. All data recorded were de-identified, and responses were anonymous to the research team.

### Study design

Respondents were administered a bespoke online survey which consisted of socio-demographic and medical history questions, a generic preference-based HRQoL measure (original and modified EQ-5D-5L), and COVID-19-related questions. The study was given a favourable ethical opinion from an independent reviewer working under the auspices of the Association of Research Managers and Administrators on 7th November 2021.

#### Socio-demographic and COVID-19-related questions

Respondents were asked about their socio-demographic and medical history details. They also completed questions assessing the impact of COVID-19 and long COVID (if applicable) on their health as well as how lockdowns and restrictions imposed during the COVID-19 pandemic affected their life. In addition, productivity losses were captured by the average number of working hours participants reported to have missed per week due to COVID-19/long COVID symptoms.

In the survey, long COVID was defined according to the National Institute for Health and Care Excellence (NICE) as symptoms which cannot be explained by an alternative diagnosis or condition and which lasted or developed 12 weeks beyond the initial COVID-19 infection [6, 11].

#### EQ-5D-5L

The EQ-5D-5L is a well-established generic, preference-based instrument that is commonly used to measure HRQoL across a variety of conditions [12]. It evaluates patients’ HRQoL on five dimensions (i.e., mobility, self-care, usual activities, pain/discomfort, and anxiety/depression), each being characterised by five levels of severity (i.e., no, slight, moderate, severe, and extreme problems). For a UK population, each health profile described by the EQ-5D-5L yields a utility value anchored on a scale where 1 represents full health and 0 represents death. States worse than dead (i.e., < 0) are possible.

The wording of the EQ-5D-5 Linstrument was amended (see Table S2 in the supplementary material) to measure respondents’ HRQoL retrospectively at various timepoints relevant to the course of a COVID-19 infection (similar to [13]). Therefore, respondents were required to recall their self-assessed HRQoL at the following (qualitative) timepoints: before having COVID-19, during the acute phase of COVID-19, and during long COVID (applicable only for the respondents who reported to have had and recovered from long COVID). The EuroQol Group approved the use and modification of the EQ-5D-5L on 21st December 2021.

In addition, all respondents completed the original EQ-5D-5L instrument which captured respondents’ current health at the time of survey completion which provided an EQ-5D-5L estimate for either post-COVID-19 (i.e., after recovering from all COVID-19-related illnesses) or long COVID (i.e., current long COVID), depending on the respondent’s current disease status.

### Data analysis

#### Data quality checks

Data quality checks included identifying ‘speeder’ participants defined as those completing the survey faster than the predefined cut-off (i.e., one third of the median completion time across all respondents). As responses from such recipients might have been less robust, the decision to exclude any such respondents was set a priori. In addition, inconsistent responses were excluded from participants who reported that their current health was better or had returned to COVID-19 levels, while their EQ-5D-5L utility score post-COVID-19 was lower than the EQ-5D-5L utility score during acute COVID-19.

### Statistical analysis

#### Descriptive statistics

Respondents’ socio-demographic and medical details, COVID-19-related data and HRQoL were summarised using averages and frequencies, as well as measures of variability, such as standard deviation (SD). EQ-5D-5L responses were converted into utility scores by applying the crosswalk algorithm which maps the EQ-5D-5L to the EQ-5D-3L using the UK value set [14], as per NICE recommendation [15].

#### Mixed-effects model regression analysis

A multilevel (or hierarchical) mixed-effects model (e.g., [16]). with a random effect at individual level was performed to capture the impact of a COVID-19 diagnosis on HRQoL to adjust the changes in HRQoL from baseline (i.e., reference category pre-COVID-19) for external factors and to capture between-respondent variation (as random intercept). A range of variables likely to impact HRQoL over time were explored as covariates in the regression model. These included socio-demographic characteristics, economic factors, medical history, and lockdown experiences. Statistical significance was designated at *p* ≤ 0.05.

The best-performing model was selected based on goodness-of-fit measures including the Akaike and Bayesian information criteria (AIC and BIC), for which a smaller value indicates a better model fit [17]. Additional details on the model specifications can be found in the supplementary material (technical appendix).

### Sensitivity analyses

Several sensitivity analyses were performed to assess the robustness of results when varying some of the model specifications and included (i) testing for presence of recall bias by including the time since the positive COVID-19 test result as covariate in the regression analysis [18], (ii) using the EQ-5D visual analogue scale (VAS) as dependent variable.

All statistical analyses were conducted using Stata® version 15.1 (StataCorp LP, College Station, TX, USA).

## Results

### Survey sample size

The total sample consisted of 406 participants reporting a positive COVID-19 test between December 2020 and January 2022, including:

1. 300 adults with mild-to-moderate COVID-19 who had not been hospitalised for COVID-19 in the acute phase (non-hospitalised adult sample), of which:

- 167 (55.7%) were considered to be at high risk for severe COVID-19 due to underlying comorbidities/risk factors (for details, see Table S3 in the supplementary material);
- 53 adults who had been hospitalised for COVID-19 in the acute phase (hospitalised adult sample),
- 53 adolescents with mild-to-moderate COVID-19 who had not been hospitalised for COVID-19 in the acute phase (adolescent sample).

The average time for completing the survey was 12.2 min (median: 9 min, interquartile range: 6.4–12.9 min). No respondents were excluded due to taking less time to complete the survey than the predefined cut-off.

However, 88 respondents (21%) were excluded from the base-case analysis because they reported their health to be the same/better compared to pre-COVID-19 levels, while a comparison on the individual-level revealed that their post-COVID-19 EQ-5D-5L utility score was lower than their pre-COVID-19 value.

### Descriptive analysis

The mean age (SD) for adults in the non-hospitalised and hospitalised samples was 48 (15) and 50 (13) years, respectively, whereas the mean age in the adolescent sample was 13 (1) years. Across all samples, most respondents were residents of England and reported their ethnicity as White. Additional information on respondent characteristics can be found in Table 1.

**Table 1:** Socio-demographic characteristics across all samples (non-hospitalised adults, hospitalised adults, and adolescents)

Characteristics	Adult non-hospitalised sample (*n* = 236)		Adult hospitalised sample (*n* = 42)		Adolescent non-hospitalised sample (*n* = 42)	
	**n**	**%**	**n**	**%**	**n**	**%**
***Socio-demographic characteristics***						
**Age (years): 12 + years old**						
Mean (SD) Median (IQR) Age (years): 16–64 years old Age (years): +65 years old	48.0 (15.3) 48.0 (36.5–62.0) 189 47	- - 80.0 19.9	49.7 (12.9) 40.5 (42.0–60.0) 35 7	- - 83.3 16.7	13.2 (1.1) 13.0 (12.0–14.0) - -	- - - -
**Sex**						
Women Men Prefer not to say	125 110 1	53.0 47 0.4	20 22 -	47.6 52.4 -	15 27 -	35.7 64.3 -
**Place of residence**						
England Scotland Wales Northern Ireland	219 8 7 2	92.8 3.4 3.0 0.9	40 - 1 1	95.2 - 2.4 2.4	41 1 - -	97.6 2.4 - -
**Ethnicity**						
White Asian/Asian British Black/Black Caribbean/African Mixed/multiple ethnic groups Other Prefer not to say	206 12 5 9 2 2	87.3 5.1 2.1 3.8 0.9 0.9	32 4 1 4 1 -	76.2 9.5 2.4 9.5 2.4 -	35 6 1 - - -	83.3 14.3 2.4 - - -
**Education**						
University degree College Secondary/high school Prefer not to say	84 70 77 5	35.9 29.7 32.6 2.1	24 7 11 -	57.1 16.7 26.2 -	NA NA NA NA	NA NA NA NA
**Current employment status**						
Working full-time (35 + hours/week) Working part-time (< 35 h/week) Unemployed Maternity/paternity leave Retired Student Other	112 46 13 3 47 6 9	47.5 19.5 5.5 1.3 19.9 2.5 3.8	25 9 1 - 6 - 1	59.5 21.4 2.4 - 14.3 - 2.4	NA NA NA NA NA NA NA	NA NA NA NA NA NA NA
**Working hours (per week) pre-pandemic [mean (SD)]** Working full-time (35 + hours/week)^a^ Working part-time (< 35 h/week)	22.1 (14.9) 35.0 10.0 (12.41)	NA NA NA	25.9 (13.2) 35.0 14.3 (14.0)	NA NA NA	NA NA NA	NA NA NA
**Household members**						
Children (15 years or younger) Adults (16 years or older) Medically vulnerable people One-person household	56 72 22 86	23.7 30.5 9.3 36.4	5 10 5 22	11.9 23.8 11.9 52.4	NA NA NA NA	NA NA NA NA

Abbreviations: IQR, interquartile range; SD, standard deviation; n, number of observations; NA, not applicable (education, employment, and household related data were not collected for the adolescent sample)

^a^ A mean (SD) could not be calculated because information on number of hours > 35 was captured using a single category of ‘35+’ in the survey

In terms of respondents with comorbidities/risk factors, there were 57% and 81% in the non-hospitalised and hospitalised adult samples, respectively. No comorbidities/risk factors were reported in the adolescent sample. In the non-hospitalised adult sample, the most frequently reported comorbidities/risk factors were obesity (26%), smoking (15%) and hypertension (15%) which were similar to the national averages (obesity: 28%, smoking: 16%, hypertension: 12% [19]). The most frequently reported comorbidity in the hospitalised adult sample was ‘told by doctor to be at increased risk from COVID-19’ (43%), followed by obesity (19%).

Table 2 presents respondents’ COVID-19-related characteristics which were found to be similar across samples. In addition, most respondents experienced symptoms during the acute phase of the disease. In the adult samples, the most frequently reported symptom was low energy/tiredness (64% and 88% in the non-hospitalised and hospitalised adult samples, respectively), while sore throat was the most reported symptom among adolescent respondents (50%). Most respondents experienced symptoms associated with long COVID, having either recovered from it or still experiencing symptoms at the time they completed the survey. Across all samples, the most frequently experienced symptom during long COVID was tiredness/fatigue (39%, 40%, and 31% in the non-hospitalised adult, hospitalised adult and adolescent samples, respectively).

Half of respondents in the non-hospitalised adult sample and one third of adolescents reported to have received no treatment for their COVID-19 symptoms during the acute phase. Among those who reported receiving treatment, over-the-counter medication was the most common (32% and 52%, respectively).

The majority of adult respondents (%) had been vaccinated against COVID-19 at the time of completing the survey (i.e., after their infection), while less than a third of adolescent respondents had received a COVID-19 vaccine after their infection (21%) (Table 1). Most respondents in the non-hospitalised adult (56%) and adolescent (84%) samples reported that their current health was about the same compared to pre-COVID-19. In the hospitalised sample, 45% of respondents reported that their current health was worse than before testing positive for COVID-19, whereas 26% reported that their current health was much better compared to pre-COVID-19.

**Table 2:** COVID-19 and economic characteristics across all samples (non-hospitalised adults, hospitalised adults, and adolescents)

Characteristics	Adult non-hospitalised sample (*n* = 236)		Adult hospitalised sample (*n* = 42)		Adolescent non-hospitalised sample (*n* = 42)	
	**n**	**%**	**n**	**%**	**n**	**%**
***COVID-19 related characteristics***						
**Positive test result for COVID-19**						
December 2020 January 2021 February 2021 March 2021 April 2021 May 2021 June 2021 July 2021 August 2021 September 2021 October 2021 November 2021 December 2021 January 2022	58 56 31 25 7 4 6 12 14 5 14 4 - -	24.6 23.7 13.1 10.6 3.0 1.7 2.5 5.1 5.9 2.1 5.9 1.7 - -	5 13 1 2 2 2 1 2 2 7 1 2 2 -	11.9 31.0 2.4 4.8 4.8 4.8 2.4 4.8 4.8 16.7 2.4 4.8 4.8 -	1 1 3 1 - - - - 4 6 2 14 8 2	2.4 2.4 7.1 2.4 - - - - 9.5 14.3 4.8 33.3 19.1 4.8
**Experienced symptoms during acute phase of COVID-19**						
Yes No	208 28	88.1 11.9	40 2	95.2 4.8	42 -	100.0 -
**Experienced long COVID**						
Yes, previously Yes, currently No Do not know	101 46 86 3	43.0 20.04 36.4 1.3	28 11 2 1	66.7 26.2 4.8 2.4	29 5 7 1	69.1 11.9 16.7 2.4
**Hospitalisation (since December 2020)**						
Due to COVID-19 (during acute COVID-19) Due to COVID-19 (after acute COVID-19) Due to an existing chronic condition Due to other reasons	- 5 5 7	- 29.4 29.4 41.2	42 1 2 -	100.0 2.4 4.8 -	- 2 1 -	- 66.7 33.3 -
**Supplementary oxygen (conditional on hospitalisation for COVID-19)**						
Yes No	2 3	40.0 60.0	37 3	92.5 7.5	2 -	100.0 -
**COVID-19 vaccination (after COVID-19 infection)**						
Yes No	185 51	78.4 21.6	30 12	71.4 28.6	12 33	21.4 78.6
**NHS treatment postponed during COVID-19 pandemic**						
Yes No Do not know	37 69 5	33.3 62.2 4.5	10 9 1	50.0 45.0 5.0	- 1 1	- 50.0 50.0
**Daily activities restricted due to measures/lockdown**						
Very much Much Somewhat Little Very little Do not know	43 47 89 31 26 -	18.2 19.9 37.7 13.1 11.0 -	14 6 6 14 2 -	33.3 14.3 14.3 33.3 4.8 -	6 5 17 11 2 1	14.3 11.9 40.5 26.2 4.8 2.4
**Struggled to adjust to measures/lockdown**						
Very much Much Somewhat Little Very little Not at all	37 38 72 32 33 24	15.7 16.1 30.5 13.6 14.0 10.2	11 10 8 6 5 2	26.2 23.8 19.1 14.3 11.9 4.8	8 6 14 10 4 -	19.1 14.3 33.3 23.8 9.5 -
**Today’s health compared to pre-COVID-19**						
Much worse Worse About the same Better Much better Do not know	8 65 132 13 12 6	3.4 27.5 56.0 5.5 5.1 2.5	4 19 6 2 11 -	9.5 45.2 14.3 4.8 26.2 -	- 2 37 1 1 1	- 4.8 88.1 2.4 2.4 2.4
***Economic characteristics***						
**Productivity loss due to COVID-19 (average working hours missed per week) [mean (SD)]**						
Acute phase Full-time working (35 + hours/week) ^a^ Part-time working (< 35 h/week) Long COVID Full-time working (35 + hours/week) ^a^ Part-time working (< 35 h/week)	17.2 (15.8) 26.0 (14.3) 17.2 (15.8) 5.1 (10.8) 7.7 (13.1) 5.1 (10.8)	- - - - - -	20.9 (15.5) 28.5 (12.6) 209 (15.5) 9.9 (13.4) 11.3 (15.3) 9.39(13.4)	- - - - - -	NA NA NA NA NA NA	NA NA NA NA NA NA

Abbreviations: SD, standard deviation; n, number of observations; NA, not applicable (working hours were not collected for the adolescent sample)

^a^ A mean (SD) could not be calculated because information on number of hours > 35 was captured using a single category of ‘35+’ in the survey

Table 3 summarises the changes in HRQoL reported by respondents at different timepoints. Mean EQ-5D-5L scores reported across the non-hospitalised adult, hospitalised adult and adolescent samples were lower during the acute phase of COVID-19 (0.62, 0.38, and 0.72, respectively) and long COVID (0.70, 0.54, and 0.85, respectively) compared to pre-COVID-19 levels (0.82, 0.81, and 0.84, respectively). Across all samples, respondents reported the lowest EQ-5D-5L utilities while being in the acute phase of COVID-19 (0.62, 0.38 and 0.73). Among non-hospitalised adults, the mean EQ-5D-5L score post-COVID-19 (0.84) was similar compared to the pre-COVID-19 score (0.81). However, this trend was not observed for hospitalised adults and adolescents who on average reported higher EQ-5D-5L utilities post-COVID-19 (0.86 and 0.95) compared to pre-COVID-19 (0.81 and 0.84).

**Table 3:** Mean (unadjusted) EQ-5D-5 L utility scores for each timepoint relevant to a COVID-19 infection across all samples (non-hospitalised adults, hospitalised adults, and adolescents)

Health-related quality of life scores	Adult non-hospitalised sample (*n* = 236)				Adult hospitalised sample (*n* = 42)				Adolescent non-hospitalised sample (*n* = 42)			
	**n**	**%**	**Mean**	**SD**	**n**	**%**	**Mean**	**SD**	**n**	**%**	**Mean**	**SD**
***EQ-5D utility score***												
Pre-COVID-19 During COVID-19 (acute phase) During long COVID Post-COVID-19	236 236 147 190	- - 62.3 81.0	0.82 0.62 0.70 0.84	0.25 0.35 0.26 0.22	42 42 39 31	- - 92.8 73.8	0.81 0.38 0.54 0.86	0.22 0.32 0.28 0.17	42 42 34 37	- - 81.0 88.0	0.84 0.73 0.85 0.95	0.28 0.26 0.23 0.15
***EQ-5D VAS score***												
Pre-COVID-19 During COVID-19 (acute phase) During long COVID Post-COVID-19	236 236 147 190	- - 62.3 81.0	75.44	20.4 25.2 23.0 18.7	42 42 39 31	- - 92.8 73.8	80.3 40.5 51.9 80.0	19.1 23.8 24.7 18.8	42 42 34 37	- - 81.0 88.0	88.5 70.3 83.3 89.6	11.02

Abbreviations: SD, standard deviation; n, number of observations.

### Multivariable analysis

Table 4 reports the results from the best-fit adjusted mixed-effects model (*N* = 320).

Compared to pre-COVID-19, the EQ-5D-5L scores were statistically significantly lower during both the acute phase of COVID-19 and while experiencing long COVID (β=-0.080, *p* < 0.001, and β=-0.072, *p* < 0.001, respectively).

Being hospitalised during the acute phase of COVID-19 had an additional negative impact on EQ-5D-5L during both acute COVID-19 and long COVID (β=-0.147, *p* = 0.026, and β=-0.186, *p* < 0.001, respectively). In addition, adolescents had statistically significantly higher EQ-5D-5L post-COVID-19 compared to pre-COVID-19 (β = 0.116, *p* < 0.001).

Moreover, the findings indicate statistically significant associations between the EQ-5D-5L utility scores and certain symptoms experienced during acute COVID-19. General pain and chills were negatively correlated with respondents’ EQ-5D-5L utilities (β=-0.142, *p* < 0.001, and β=-0.134 *p* < 0.001, respectively). None of the symptoms experienced during long COVID was statistically significantly associated with the EQ-5D-5L utility scores.

Regarding socio-demographic factors, the analysis found no evidence of associations between the EQ-5D-5L scores and age, sex, or ethnicity, neither as standalone covariates nor as interaction terms with COVID-19 timepoints. However, respondents whose highest level of education was secondary school had significantly lower EQ-5D-5L scores compared to respondents who had a university degree (β=-0.073, *p* = 0.007). Productivity losses experienced during acute COVID-19 were also found to be negatively correlated with EQ-5D-5L utilities (β=-0.002, *p* = 0.041).

Patients with comorbidities/risk factors that were associated with high risk for severe COVID-19 during the acute phase were found to have a lower HRQoL. These included diabetes (β=-0.224, *p* = 0.007), smoking (β=-0.117, *p* = 0.004) and hypertension (β=-0.103, *p* = 0.006). Overall, interactions between COVID-19 timepoints and comorbidities were not statistically significant.

**Table 4:** HRQoL - results of the mixed-effects model for all samples after excluding inconsistent responses (*N* = 320)

EQ-5D-5L utility score	β		Robust SE	*p*	95% CI	
**Time**						
Pre-COVID-19	Ref					
Acute COVID-19	-0.080	0.024		0.001	-0.128	-0.032
Long COVID	-0.072	0.016		< 0.001	-0.103	-0.042
Post-COVID-19	0.001	0.009		0.878	-0.016	0.018
**Hospitalisation (during first 4 weeks after COVID-19 diagnosis)**	-0.020	0.041		0.625	-0.101	0.060
**Hospitalisation interactions**						
Hospitalised x acute COVID-19	-0.147	0.066		0.026	-0.276	-0.018
Hospitalised x long COVID	-0.186	0.053		< 0.001	-0.289	-0.082
Hospitalised x post COVID-19	0.059	0.039		0.126	-0.017	0.135
**Adolescent**	-0.074	0.047		0.114	-0.167	-0.018
**Adolescent interactions**						
Adolescent x acute COVID-19	-0.020	0.041		0.627	-0.101	0.061
Adolescent x long COVID	0.086	0.047		0.068	-0.006	0.178
Adolescent x post COVID-19	0.116	0.032		< 0.001	0.053	0.180
**Acute symptoms**						
General pain	-0.142	0.040		< 0.001	-0.220	-0.063
Chills	-0.134	0.034		< 0.001	-0.201	-0.067
**Long COVID symptoms**						
Difficulty thinking	-0.050	0.036		0.162	-0.120	-0.020
**Education**						
University	Ref					
College	-0.012	0.029		0.688	-0.069	0.045
School	-0.073	0.027		0.007	-0.126	-0.020
Other	-0.014	0.118		0.906	-0.246	0.218
**Employment**						
Full-time	Ref					
Part-time	-0.019	0.028		0.502	-0.074	0.036
Unemployed	-0.281	0.070		< 0.001	-0.418	-0.144
Retired	0.014	0.030		0.635	-0.045	0.074
Student	-0.194	0.087		0.026	-0.364	0.023
Other	-0.132	0.069		0.056	-0.268	0.003
**Productivity losses (hours missed per week)**						
Acute phase	-0.002	0.001		0.041	-0.004	0.001
**Household status**						
Including up to 15 years old	0.053	0.027		0.051	-0.001	0.106
Including 16 years old or older	0.001	0.024		0.991	-0.047	0.047
Including vulnerable people	-0.061	0.031		0.052	-0.122	0.001
**Comorbidities**						
Diabetes	-0.224	0.083		0.007	-0.386	-0.062
Smoking	-0.117	0.040		0.004	-0.196	-0.038
Hypertension	-0.103	0.037		0.006	-0.176	-0.030
**Constant**	0.922	0.025		< 0.001	0.874	0.971
**Number of observations**	1411					
**Goodness-of-fit statistics**						
AIC	-273.4					
BIC	-112.8					

Abbreviations: β, coefficient; AIC, Akaike information criteria; BIC, Bayesian information criteria; CI, confidence intervals; p, *p* value; Ref, reference; SE, standard error

### Sensitivity analyses

The first sensitivity analysis did not find evidence of recall bias as the coefficient for time since the positive test was not statistically significant.

The second sensitivity analysis, including the EQ-VAS as the dependent variable in the mixed-effects model regression, generated similar trends to the ones that had been found in the primary analysis (see Table S4 in the supplementary material). However, in this sensitivity analysis, adolescents reported on average significantly higher EQ-5D-5L utilities compared to adults (β = 9.4, *p* < 0.01). In addition, adolescents reported higher EQ-5D-5L utilities during long COVID compared to their pre-COVID-19 value (β = 7.7, *p* = 0.026).

## Discussion

### Impact of COVID-19 on HRQoL

The aim of this study was to determine how HRQoL changes over time among individuals with mild-to-moderate COVID-19 in the UK. Since early studies on COVID-19 focused on capturing clinical outcomes among hospitalised patients, this study is one of the first to capture changes in HRQoL associated with mild-to-moderate COVID-19 over time [20].

In line with previous findings [21, 22], our results suggest that COVID-19 had a negative impact on HRQoL. However, other than Di Fusco et al. (2022) [21], we found evidence that HRQoL among UK patients with mild-to-moderate COVID-19 returned to pre-COVID-19 levels after recovering from an acute infection and/or long COVID. In our results, there was a clear pattern among non-hospitalised adults where acute COVID-19 had the strongest negative association with HRQoL, followed by long COVID. In contrast, among hospitalised adults, the difference in the association between HRQoL and acute and long COVID-19, respectively, seemed more pronounced, and long COVID had a bigger negative impact on HRQoL than acute COVID-19. Nevertheless, results from the mixed-effects model suggested that reductions in average HRQoL for both the acute phase of COVID-19 and long COVID were similar, confirming previous findings [22].

To the best of our knowledge, there are currently no published studies reporting estimates of minimally important differences for EQ-5D in patients with COVID-19, therefore we cannot assess the clinical significance of our findings. However, a published review reported estimates of minimally important difference for the EQ-5D using the UK 3L scoring algorithm to range from 0.03 to 0.52 [23]. Based on these estimates, the coefficients derived from the mixed model in the present study, which represent utility decrements for having acute COVID-19 (e.g., β=-0.08) and long COVID (β=-0.072), can be interpreted as clinically meaningful. More research on the minimally important difference for EQ-5D in patients with COVID-19 will further add to the evidence generated in our study.

We highlight that respondents with existing comorbidities/risk factors for severe COVID-19, including diabetes and hypertension, and respondents self-reporting as current smokers were found to have a significantly lower general HRQoL, irrespective of COVID-19-related impacts, compared to those for whom these factors were absent. The higher comorbidity prevalence among hospitalised participants likely increased the risk of hospitalisation in this group, since chronic conditions, such as diabetes, hypertension and being overweight/obese as well as risk factors including smoking, have been found to increase the risk of morbidity and mortality as well as the risk of hospitalisation for COVID-19 [1, 24]. HRQoL estimated for long COVID among hospitalised respondents was found to be lower than the HRQoL during the acute phase, suggesting that those with underlying conditions might be more affected by COVID-19 and associated long-term symptoms, supporting previous findings [20, 25]. This might be explained by the fact that these patients were experiencing a prolonged impact of the infection beyond the acute phase or that their existing chronic condition(s) may have increased the negative impact of common symptoms or caused more symptoms associated with each phase of COVID-19 (see e.g., [9, 20]). This would further support previous findings for a prolonged effect and reduced general health in patients who had been hospitalised with COVID-19 after discharge and up to one year after infection [9, 10].

Adolescents reported on average higher HRQoL compared to adult respondents for all timepoints. However, no statistically significant differences in EQ-5D-5L utility scores over time were found in the adjusted analyses, except for a higher HRQoL score among adolescents for the post-COVID-19 value compared to pre-COVID-19 levels. While this finding might suggest that adolescents found it easier to report their current (i.e., post-COVID-19) HRQoL compared to recalling their pre-COVID-19 HRQoL, the analysis found no evidence for a recall bias among adolescent respondents.

### Strength and limitations

#### Strengths

A major strength of this study is capturing EQ-5D-5L estimates over time, describing changes in HRQoL between pre- and post-COVID-19 timepoints and providing quasi-longitudinal data for a relatively large sample. In the absence of longitudinal studies describing the impact of COVID-19 on HRQoL, this approach allowed new insights into how people with COVID-19 perceived their HRQoL to have changed since before testing positive for COVID-19. Findings from this study provide HRQoL data covering earlier stages of the pandemic, thereby providing valuable information which supplements available clinical outcome data from that time. Mixed-effects models with robust standard errors accounted for heterogeneity between respondents and potential differences in characteristics that remained unobserved and could therefore not enter the HRQoL regression model directly.

#### Limitations

Although we did not find evidence of recall bias, errors in reporting are nonetheless possible. Pooling data from all respondents provided more statistical power when estimating the impact of COVID-19 on HRQoL over the course of the disease. However, more detailed results for specific symptoms and additional differences between specific subgroups could not be assessed due to the sample size. In particular, potential differences among hospitalised patients and adolescents compared to the non-hospitalised adult sample could not be quantified and their impact was only estimated through the average effect found between the different groups (i.e., non-hospitalised adult sample vs. hospitalised/adolescent sample). Based on the chosen recruitment strategy, the study relied on participants who self-reported a positive COVID-19 test result and who had received a monetary compensation for completing the survey. We acknowledge that this is a limitation of the study that we could not control for. However, before being selected to participate into the study, participants were asked several open inclusion/exclusion questions that aimed to select a sufficiently large sample, allowing for the estimation of robust results. In addition, we recognize that the results may suffer from sample selection bias, such as the high percentage of participants who reported to have experienced long COVID who may have over/underestimated HRQoL at the associated timepoint. However, long COVID remains under-researched and evidence of its impact on patients’ HRQoL is limited. Nevertheless, additional action plans are currently being developed, recognising the impact it has on patients [26].

### Electronic supplementary material

Below is the link to the electronic supplementary material.

Supplementary Material 1

## Data Availability

The data are available upon request.
