# Supplementary material for: Health-related quality of life in mild-to-moderate COVID-19 in the UK: a cross-sectional study from pre- to post-infection
Source: Health Qual Life Outcomes. 2024 Jan 30;22:12. doi: 10.1186/s12955-024-02230-5 (PMC10826014; doi:10.1186/s12955-024-02230-5)
Supplement: Supplementary file 1 — Supplementary Material 1 [file 12955_2024_2230_MOESM1_ESM.docx]

# **Supplementary material**

## **Tables**

**Table S 1. Inclusion and exclusion criteria across all samples**

| Criteria | Subsets of study population | |
| --- | --- | --- |
| Inclusion criteria | **Non-hospitalised adults (n=300)**  **+**  **adolescent sample (n=53)** | **Hospitalised adults sample (n=51)** |
| Be either an adult (≥16 years of age) or adolescent (12-15 years of age) living in the UK | ۷ | ۷ |
| Have a self-reported diagnosis for COVID-19, by receiving a positive COVID-19 test result between 4 weeks and up to 12 months prior to the completion of the study survey | ۷ | ۷ |
| Have received treatment or monitoring for COVID-19 within an outpatient setting only (i.e., not being hospitalised) during the first four weeks after a positive COVID-19 test | ۷ | × |
| Be able to read and understand English | ۷ | ۷ |
| Have access to internet and a computer, laptop, or tablet | ۷ | ۷ |
| Be able and willing to complete an online questionnaire | ۷ | ۷ |
| Have given consent (≥16 years of age) or consent on behalf of the child/young person (12-15 years of age) indicating that they have been informed of all pertinent aspects of the study and that they agree to take part in the survey. | ۷ | ۷ |
| Exclusion criteria | **Non-hospitalised adults (n=300)**  **+**  **adolescent sample (n=53)** | **Hospitalised adults sample (n=51)** |
| Have a COVID-19 infection more than once | ۷ | ۷ |
| Have a positive COVID-19 test after receiving at least one vaccination dose for COVID-19 | ۷ | ۷ |
| Have taken part in any clinical trial related to COVID-19 vaccines or anti-viral treatment | ۷ | ۷ |
| Abbreviations: ۷- Criterion applies; × - Criterion does not apply | | |

**Table S 2. Amended wording of the EQ-5D-5L approved by EuroQol**

| Timepoints during COVID-19 infection | Amended wording of the EQ-5D-5L |
| --- | --- |
| Pre-COVID-19 | You will be asked to complete a short questionnaire about your health **before the onset of any COVID-19 related symptoms** or **before you tested positive for COVID-19**. |
| During acute COVID-19 | You will be asked to complete a short questionnaire about **your health while you were experiencing the worst symptoms** in the acute phase of COVID-19. |
| During long COVID | You will be asked to complete a short questionnaire about your health **while you were experiencing symptoms associated with long COVID** before you recovered. |

**Table S 3. High-risk criteria for severe acute COVID-19**

| High-risk criteria for severe acute COVID-19 |
| --- |
| - Be at least 65 years of age - Be overweight and obese:   - 1. Overweight (defined as a body mass index (BMI) > 25 kg/m2 but < 30 kg/m2)     2. Obesity (BMI ≥30 kg/m2 but < 40 kg/m2)     3. Severe obesity (BMI of ≥40 kg/m2) - Be a current smoker (cigarette smoking within the past 30 days) and history of at least 100 lifetime cigarettes - Have immune deficiencies / immunocompromised state:   - 1. Primary immunodeficiency is caused by genetic defects that can be inherited.     2. Prolonged use of corticosteroids or other immune weakening medicines can lead to secondary or acquired immunodeficiency - Have an HIV infection - Have a Solid organ or blood stem cell transplant - Have a chronic lung disease - Have COPD, emphysema and chronic bronchitis - Have asthma (moderate-to-severe):   - 1. Other lung disease (interstitial lung disease, pulmonary fibrosis, pulmonary hypertension)     2. Cystic fibrosis with or without lung or other solid organ transplant - Have a known diagnosis of hypertension - Have cardiovascular disease (CVD), defined as history of any of the following: myocardial infarction, stroke, TIA, HF, angina with prescribed nitroglycerin, CABG, PCI, carotid endarterectomy, cerebrovascular disease, and aortic bypass - Have Type 1 or Type 2 diabetes mellitus - Have CKD at any stage - Have sickle cell disease - Have neurodevelopmental disorders (e.g., dementia, cerebral palsy, Down’s syndrome) or other conditions that confer medical complexity (e.g., genetic or metabolic syndromes and severe congenital anomalies) - Have an active cancer diagnosis, other than localized skin cancer, including those requiring treatment as long as the treatment is not among the prohibited medications that must be administered/continued during the trial period - Have a medical-related technological dependence (e.g., CPAP [not related to COVID-19]). - Have a liver disease:   - 1. alcohol-related liver disease     2. non-alcoholic fatty liver disease     3. cirrhosis - Be pregnant or recent pregnant (42 days following end of pregnancy) - Have substance abuse disorders:   - 1. Alcohol use disorder     2. Opioid use disorder     3. Cocaine use disorder |

**Table S 4. Sensitivity analysis 2: HRQoL - results from the mixed-effects model for all samples using the EQ-5D VAS as dependent variable (N=404).**

| EQ-5D-5L VAS score | β | Robust SE | p | 95% CI | | |
| --- | --- | --- | --- | --- | --- | --- |
| Time | | | | | | |
| Pre COVID-19 | Ref |  |  | |  |  |
| Acute COVID-19 | -14.046 | 1.866 | <0.001 | | -17.704 | -10.388 |
| Long COVID | -13.873 | 1.665 | <0.001 | | -17.136 | -10.609 |
| Post COVID-19 | -2.478 | 0.883 | 0.005 | | -4.209 | -0.747 |
| Hospitalisation (during first 4 weeks after COVID-19 diagnosis) | 2.780 | 3.016 | 0.357 | | -3.131 | 8.692 |
| Hospitalisation interactions | | | | | | |
| Hospitalised x acute COVID-19 | -8.917 | 4.245 | 0.036 | | -17.237 | -0.596 |
| Hospitalised x long COVID | -11.786 | 4.093 | 0.004 | | -19.808 | -3.764 |
| Hospitalised x post COVID-19 | 2.760 | 2.823 | 0.328 | | -2.772 | 8.292 |
| Adolescent | 9.410 | 2.531 | <0.001 | | 4.449 | 14.371 |
| Adolescent interactions | | | | | | |
| Adolescent x acute COVID-19 | -4.091 | 3.389 | 0.227 | | -10.734 | 2.552 |
| Adolescent x long COVID | 7.713 | 3.472 | 0.026 | | 0.907 | 14.519 |
| Adolescent x post COVID-19 | 2.937 | 1.798 | 0.102 | | -0.588 | 6.461 |
| Acute symptoms | | | | | | |
| General pain | -10.183 | 2.700 | <0.001 | | -15.475 | -4.890 |
| Chills | -13.434 | 2.401 | <0.001 | | -18.141 | -8.726 |
| Long COVID symptoms | | | | | | |
| Difficulty thinking | -6.341 | 2.786 | 0.023 | | -11.801 | -0.880 |
| Education | | | | | | |
| University | Ref |  |  | |  |  |
| College | 1.020 | 2.057 | 0.620 | | -3.012 | 5.051 |
| School | -2.453 | 2.047 | 0.231 | | -6.465 | 1.559 |
| Other | -1.554 | 7.904 | 0.844 | | -17.045 | 13.937 |
| Employment | | | | | | |
| Full-time | Ref |  |  | |  |  |
| Part-time | -2.620 | 2.220 | 0.238 | | -6.970 | 1.731 |
| Unemployed | -13.897 | 4.739 | 0.003 | | -23.185 | -4.609 |
| Retired | 1.892 | 2.602 | 0.467 | | -3.208 | 6.992 |
| Student | 0.506 | 4.479 | 0.910 | | -8.273 | 9.286 |
| Other | -7.331 | 5.099 | 0.150 | | -17.325 | 2.662 |
| Productivity losses (hours missed per week) | | | | | | |
| Acute phase | -0.119 | 0.066 | 0.073 | | -0.249 | 0.011 |
| Household status | | | | | | |
| Including up to 15 years old | 5.398 | 1.858 | 0.004 | | 1.757 | 9.039 |
| Including 16 years old or older | -0.398 | 1.758 | 0.821 | | -3.845 | 3.048 |
| Including vulnerable people | -4.317 | 2.189 | 0.049 | | -8.608 | -0.026 |
| Comorbidities | | | | | | |
| Diabetes | -11.610 | 4.278 | 0.007 | | -19.995 | -3.226 |
| Smoking | -7.545 | 2.703 | 0.005 | | -12.842 | -2.247 |
| Hypertension | -5.601 | 2.425 | 0.021 | | -10.355 | -0.848 |
| Constant | 80.401 | 2.110 | <0.001 | | 76.266 | 84.537 |
| Number of observations | 1411 |  |  | |  |  |
| Abbreviations: β, coefficient; AIC, Akaike information criteria; BIC, Bayesian information criteria; CI, confidence intervals; p, p value; SE, standard error. | | | | | | |

## **Technical appendix**

### **Mixed effects model**

Data were analysed using multilevel (or hierarchical) mixed-effects linear regression models (1, 2) in Stata 15.1 (Stata Corp LLC) using the *mixed* command with robust standard errors. Individual respondents were modelled as a random effect (3-5) to capture additional levels of heterogeneity between respondents and to account for correlated measurements across time for each respondent (controlling, for instance, for the fact that some individuals will always report lower or higher HRQoL than others).

Other variables for which the model estimated on impact on HRQoL were included as fixed effects as described in Equation 1.

**Equation 1. Mixed-effects model**

$${eq5d}_{ij}= \beta_{0}+ \beta_{1}X+ \beta_{2}Covid+ u_{j}+ \epsilon_{ij}$$

for *i*=1, …, I respondents and *j*=1, …, 4 (timepoints corresponding with COVID-19-related stages). The fixed portion of the model, $\beta_{0}+ \beta_{1}X+ \beta_{2}Covid$, describes the overall prediction representing the average for the sample population based on individual characteristics *X* that do not vary over time, and COVID-related factors *Covid* that capture fixed effects in the relevant COVID-19 stage(s) only. The random effect *u_j_* describes the constant intercept that shifts the population average according to the individual respondent *i*. A normal distribution was specified for the error $\epsilon_{ij}$.

### **Variable selection**

All variables included in the final mixed-effects model are described in Table 1.

**Table 1. List of final covariates included in the mixed effects model**

| Variable | Constant over time | Timepoint |
| --- | --- | --- |
| Dependent variable | | |
| EQ-5D-5L utility | No | Pre-COVID-19 (reference category)  Acute COVID-19  Long COVID  Post-COVID-19 |
| Covariates | | |
| *COVID-19-related variables* | | |
| Hospitalised (for COVID-19 during acute phase) | No | Acute COVID-19 |
| Symptoms | No | Acute COVID-19  Long COVID |
| Productivity losses | No | Acute COVID-19 |
| *Individual characteristics* | | |
| Age (baseline) | Yes |  |
| Sex | Yes |  |
| Adolescent (12-15 years) | Yes |  |
| Education | Yes |  |
| Employment status | Yes |  |
| Household status | Yes |  |
| Comorbidities/risk factors (diabetes, hypertension, smoking) | Yes |  |

Variables were chosen for inclusion in the model by a stepwise approach, considering both the goodness-of-fit measures described below and potential correlation between groups of variables. For instance, if several symptoms, that respondents reported to have experienced in either the acute or long COVID-19 phase, were found to be highly correlated (i.e., >0.4), only one covariate from that group was included in the regression analysis to account for the collinearity between covariates. The correlation matrices below (Table 2, Table 3) provide further information.

**Table 2. Correlation matrix results: symptoms during acute COVID-19**

|  | Stuf_  nose | Sore  throat | Loss_  smell | BR | Cough | Low En | MU | PA | Chest _PA | Head | Think | Dizz | Chills | FE | NA | VO | DI | Pins | SL | Mood | Me_loss | Rash | Toes | Mens |
| --- | --- | --- | --- | --- | --- | --- | --- | --- | --- | --- | --- | --- | --- | --- | --- | --- | --- | --- | --- | --- | --- | --- | --- | --- |
| Stuf_ nose | 1.00 |  |  |  |  |  |  |  |  |  |  |  |  |  |  |  |  |  |  |  |  |  |  |  |
| Sore throat | 0.63 | 1.00 |  |  |  |  |  |  |  |  |  |  |  |  |  |  |  |  |  |  |  |  |  |  |
| Loss_smell | 0.45 | 0.46 | 1.00 |  |  |  |  |  |  |  |  |  |  |  |  |  |  |  |  |  |  |  |  |  |
| BR | 0.47 | 0.51 | 0.53 | 1.00 |  |  |  |  |  |  |  |  |  |  |  |  |  |  |  |  |  |  |  |  |
| Cough | 0.51 | 0.62 | 0.59 | 0.61 | 1.00 |  |  |  |  |  |  |  |  |  |  |  |  |  |  |  |  |  |  |  |
| Low En | 0.55 | 0.59 | 0.66 | 0.67 | 0.67 | 1.00 |  |  |  |  |  |  |  |  |  |  |  |  |  |  |  |  |  |  |
| MU | 0.48 | 0.56 | 0.57 | 0.65 | 0.65 | 0.80 | 1.00 |  |  |  |  |  |  |  |  |  |  |  |  |  |  |  |  |  |
| PA | 0.40 | 0.49 | 0.49 | 0.60 | 0.52 | 0.59 | 0.63 | 1.00 |  |  |  |  |  |  |  |  |  |  |  |  |  |  |  |  |
| Chest_PA | 0.34 | 0.38 | 0.40 | 0.48 | 0.44 | 0.43 | 0.47 | 0.51 | 1.00 |  |  |  |  |  |  |  |  |  |  |  |  |  |  |  |
| Head | 0.52 | 0.59 | 0.61 | 0.58 | 0.62 | 0.71 | 0.71 | 0.56 | 0.47 | 1.00 |  |  |  |  |  |  |  |  |  |  |  |  |  |  |
| Think | 0.38 | 0.43 | 0.53 | 0.58 | 0.51 | 0.59 | 0.58 | 0.59 | 0.51 | 0.58 | 1.00 |  |  |  |  |  |  |  |  |  |  |  |  |  |
| Dizz | 0.44 | 0.46 | 0.49 | 0.60 | 0.51 | 0.56 | 0.56 | 0.54 | 0.48 | 0.60 | 0.57 | 1.00 |  |  |  |  |  |  |  |  |  |  |  |  |
| Chills | 0.37 | 0.43 | 0.52 | 0.59 | 0.56 | 0.64 | 0.67 | 0.62 | 0.49 | 0.64 | 0.60 | 0.54 | 1.00 |  |  |  |  |  |  |  |  |  |  |  |
| FE | 0.43 | 0.50 | 0.48 | 0.55 | 0.59 | 0.60 | 0.61 | 0.56 | 0.54 | 0.59 | 0.55 | 0.58 | 0.66 | 1.00 |  |  |  |  |  |  |  |  |  |  |
| NA | 0.36 | 0.40 | 0.43 | 0.50 | 0.48 | 0.48 | 0.48 | 0.46 | 0.52 | 0.51 | 0.48 | 0.53 | 0.47 | 0.53 | 1.00 |  |  |  |  |  |  |  |  |  |
| VO | 0.29 | 0.29 | 0.27 | 0.32 | 0.32 | 0.32 | 0.31 | 0.33 | 0.39 | 0.33 | 0.35 | 0.29 | 0.29 | 0.27 | 0.40 | 1.00 |  |  |  |  |  |  |  |  |
| DI | 0.29 | 0.29 | 0.36 | 0.38 | 0.35 | 0.40 | 0.41 | 0.43 | 0.40 | 0.40 | 0.43 | 0.35 | 0.41 | 0.34 | 0.40 | 0.41 | 1.00 |  |  |  |  |  |  |  |
| Pins | 0.24 | 0.32 | 0.29 | 0.43 | 0.33 | 0.34 | 0.36 | 0.43 | 0.48 | 0.34 | 0.44 | 0.45 | 0.39 | 0.38 | 0.47 | 0.34 | 0.29 | 1.00 |  |  |  |  |  |  |
| SL | 0.38 | 0.44 | 0.46 | 0.55 | 0.49 | 0.58 | 0.55 | 0.50 | 0.48 | 0.56 | 0.54 | 0.56 | 0.54 | 0.52 | 0.52 | 0.32 | 0.34 | 0.45 | 1.00 |  |  |  |  |  |
| Mood | 0.30 | 0.41 | 0.36 | 0.40 | 0.37 | 0.37 | 0.40 | 0.39 | 0.48 | 0.40 | 0.46 | 0.48 | 0.38 | 0.43 | 0.48 | 0.37 | 0.36 | 0.42 | 0.51 | 1.00 |  |  |  |  |
| Me_loss | 0.28 | 0.33 | 0.40 | 0.42 | 0.40 | 0.43 | 0.42 | 0.40 | 0.49 | 0.35 | 0.53 | 0.44 | 0.43 | 0.44 | 0.40 | 0.32 | 0.29 | 0.40 | 0.43 | 0.44 | 1.00 |  |  |  |
| Rash | 0.24 | 0.23 | 0.30 | 0.27 | 0.29 | 0.28 | 0.28 | 0.33 | 0.38 | 0.25 | 0.29 | 0.34 | 0.29 | 0.28 | 0.33 | 0.37 | 0.36 | 0.35 | 0.34 | 0.36 | 0.41 | 1.00 |  |  |
| Toes | 0.17 | 0.23 | 0.22 | 0.21 | 0.18 | 0.21 | 0.24 | 0.32 | 0.35 | 0.27 | 0.26 | 0.28 | 0.28 | 0.25 | 0.31 | 0.34 | 0.27 | 0.39 | 0.29 | 0.33 | 0.32 | 0.43 | 1.00 |  |
| Mens | 0.14 | 0.14 | 0.24 | 0.23 | 0.16 | 0.22 | 0.25 | 0.20 | 0.25 | 0.28 | 0.29 | 0.28 | 0.31 | 0.30 | 0.26 | 0.23 | 0.15 | 0.15 | 0.24 | 0.28 | 0.16 | 0.11 | 0.21 | 1.00 |
| Stuff_nose – Stuffy nose; Loss_smell – Loss of smell and taste; BR – Breathing problems; Low En – Low energy; MU – Muscle ache; PA – General pain; Chest_PA – Chest or stomach pain; Head – Headache; Think – Difficulty thinking; Dizz – Dizziness; FE – Fever; NA – Nausea; VO – vomiting; DI – Diarrhoea; Pins – Pins and needles feeling; SL – Sleep problems; Mood – Mood change; Me_loss- Memory loss; Rash – Skin rash; Toes – COVID-19 toes; Mens – Menstruation changes. | | | | | | | | | | | | | | | | | | | | | | | | |

|  | Tired | FE | PA | BR | Cough | Chest_  PA | Palp | Ch_smell | Head | Dizz | Think | Pins | SL | Mood | Me_loss | DI | MU | Rash | Toes | Mens |
| --- | --- | --- | --- | --- | --- | --- | --- | --- | --- | --- | --- | --- | --- | --- | --- | --- | --- | --- | --- | --- |
| Tired | 1.00 |  |  |  |  |  |  |  |  |  |  |  |  |  |  |  |  |  |  |  |
| FE | 0.31 | 1.00 |  |  |  |  |  |  |  |  |  |  |  |  |  |  |  |  |  |  |
| PA | 0.48 | 0.15 | 1.00 |  |  |  |  |  |  |  |  |  |  |  |  |  |  |  |  |  |
| BR | 0.44 | 0.22 | 0.35 | 1.00 |  |  |  |  |  |  |  |  |  |  |  |  |  |  |  |  |
| Cough | 0.35 | 0.32 | 0.23 | 0.27 | 1.00 |  |  |  |  |  |  |  |  |  |  |  |  |  |  |  |
| Chest_PA | 0.30 | 0.25 | 0.30 | 0.33 | 0.26 | 1.00 |  |  |  |  |  |  |  |  |  |  |  |  |  |  |
| Palp | 0.36 | 0.19 | 0.28 | 0.40 | 0.16 | 0.35 | 1.00 |  |  |  |  |  |  |  |  |  |  |  |  |  |
| Ch_smell | 0.33 | 0.14 | 0.37 | 0.32 | 0.20 | 0.22 | 0.26 | 1.00 |  |  |  |  |  |  |  |  |  |  |  |  |
| Head | 0.44 | 0.27 | 0.38 | 0.30 | 0.33 | 0.26 | 0.31 | 0.31 | 1.00 |  |  |  |  |  |  |  |  |  |  |  |
| Dizz | 0.31 | 0.23 | 0.34 | 0.34 | 0.26 | 0.26 | 0.35 | 0.17 | 0.44 | 1.00 |  |  |  |  |  |  |  |  |  |  |
| Think | 0.46 | 0.21 | 0.41 | 0.51 | 0.25 | 0.29 | 0.39 | 0.32 | 0.41 | 0.36 | 1.00 |  |  |  |  |  |  |  |  |  |
| Pins | 0.21 | 0.25 | 0.27 | 0.28 | 0.11 | 0.28 | 0.20 | 0.22 | 0.21 | 0.30 | 0.29 | 1.00 |  |  |  |  |  |  |  |  |
| SL | 0.40 | 0.17 | 0.44 | 0.37 | 0.21 | 0.25 | 0.29 | 0.32 | 0.38 | 0.28 | 0.52 | 0.30 | 1.00 |  |  |  |  |  |  |  |
| Mood | 0.39 | 0.30 | 0.29 | 0.32 | 0.17 | 0.30 | 0.32 | 0.18 | 0.19 | 0.22 | 0.51 | 0.28 | 0.45 | 1.00 |  |  |  |  |  |  |
| Me_loss | 0.29 | 0.16 | 0.23 | 0.39 | 0.13 | 0.22 | 0.32 | 0.25 | 0.27 | 0.28 | 0.43 | 0.22 | 0.26 | 0.18 | 1.00 |  |  |  |  |  |
| DI | 0.06 | 0.05 | 0.07 | 0.12 | 0.03 | 0.18 | 0.22 | 0.12 | 0.03 | -0.01 | 0.12 | -0.01 | 0.13 | 0.21 | 0.04 | 1.00 |  |  |  |  |
| MU | 0.44 | 0.24 | 0.57 | 0.32 | 0.23 | 0.39 | 0.26 | 0.30 | 0.34 | 0.33 | 0.40 | 0.33 | 0.42 | 0.25 | 0.32 | 0.09 | 1.00 |  |  |  |
| Rash | 0.07 | 0.05 | 0.07 | 0.08 | 0.03 | 0.13 | 0.05 | 0.13 | 0.03 | 0.05 | 0.13 | 0.08 | 0.14 | 0.11 | 0.04 | 0.11 | 0.15 | 1.00 |  |  |
| Toes | 0.12 | 0.06 | 0.15 | 0.20 | 0.10 | 0.15 | 0.06 | 0.16 | 0.15 | 0.21 | 0.15 | 0.20 | 0.11 | 0.06 | 0.18 | -0.00 | 0.18 | 0.14 | 1.00 |  |
| Mens | 0.10 | 0.11 | 0.07 | 0.08 | 0.03 | -0.01 | 0.11 | 0.09 | 0.08 | 0.18 | 0.13 | -0.01 | 0.14 | 0.17 | 0.10 | -0.01 | 0.10 | -0.01 | -0.00 | 1.00 |
| Tired – Tiredness; FE – Fever; PA – Pain; Chest_PA – Chest or stomach pain; Palp – Heart palpitations; Ch_smell – Change in smell or taste; Head – Headache; Dizz – Dizziness; Think – Difficulty thinking; Pins – Pins & needles feeling; SL – Sleep problems; Mood – Mood change; Me_loss – Memory loss; DI – Diarrhoea; MU – Muscle ache; Rash – Skin rash; Toes – COVID-19 toes; Mens – Menstruation changes. | | | | | | | | | | | | | | | | | | | | |

**Table 3. Correlation matrix results: symptoms during long COVID**

### **References**

1. Greene WH. Econometric Analysis: Stern School of Business, New York University; 2008.

2. Griffiths A, Paracha N, Davies A, Branscombe N, Cowie MR, Sculpher M. Analyzing Health-Related Quality of Life Data to Estimate Parameters for Cost-Effectiveness Models: An Example Using Longitudinal EQ-5D Data from the SHIFT Randomized Controlled Trial. Advances in therapy. 2017;34(3):753-64.

3. Arellano M. Panel Data Econometrics. New York: Oxford University Press; 2003.

4. Stock JH, Watson MW. Heteroskedasticity-Robust Standard Errors for Fixed Effects Panel Data Regression. Econometrica. 2008;76(1):155-74.

5. Woolridge J. Introductory econometrics: A modern approach. : Nelson Education; 2016.
